# Rate of Spontaneous Preterm Delivery Between Pregnant Women With and Without Gestational Diabetes

**DOI:** 10.7759/cureus.34565

**Published:** 2023-02-02

**Authors:** Dittakarn Boriboonhirunsarn, Sirikul Tanpong

**Affiliations:** 1 Obstetrics and Gynaecology, Faculty of Medicine Siriraj Hospital, Mahidol University, Bangkok, THA

**Keywords:** pregnancy outcomes, previous preterm birth, risk factors, gestational diabetes mellitus, spontaneous preterm delivery

## Abstract

Objective

The aim of this study is to compare the rate of spontaneous preterm delivery between gestational diabetes mellitus (GDM) and normal pregnancy. Pregnancy outcomes and associated risk factors for spontaneous preterm delivery were evaluated.

Methods

A retrospective cohort study was conducted on 120 GDM and 480 normal pregnant women. All women received GDM screening with 50-g glucose challenge test and 100-g oral glucose tolerance test at the first visit and repeated at 24-28 weeks. Data were retrieved from medical records and included baseline and obstetric characteristics, preterm risks, GDM risks, and pregnancy outcomes. Spontaneous preterm birth was defined as delivery before 37 completed weeks of gestation that had been preceded by spontaneous labor.

Results

GDM women were more likely to be *≥*30 years (p=0.032) and have previous GDM (p=0.013). Incidence of overall preterm delivery was significantly higher in GDM women (17.5% vs. 8.5%, p=0.004), as well as the incidence of spontaneous preterm delivery (15.8% vs. 7.1%, p=0.004). GDM women had less gestational weight gain (p<0.001) and were less likely to have excessive weight gain (p=0.002). GDM women were more likely to deliver large for gestational age (LGA) (p=0.02) and macrosomic infants (p=0.027). Neonatal hypoglycemia was significantly more common among GDM women (p=0.013). Multivariate analysis showed that previous preterm birth and GDM independently increased the risk of spontaneous preterm delivery (adjusted OR: 2.56, 95% CI: 1.13-5.79, p=0.024, and adjusted OR: 2.15, 95% CI: 1.2-3.84, p = 0.010, respectively).

Conclusion

GDM and previous preterm birth significantly increased the risk of spontaneous preterm delivery. GDM also increased the risk of LGA, macrosomia, and neonatal hypoglycemia.

## Introduction

Preterm delivery is defined as birth that takes place between 20 weeks to less than 37 weeks of gestational age (GA) [1]. The least GA determining the viability of the babies depends on the abilities of the pediatric team and the facilities of the hospital. Incidence of preterm delivery varied from 5% to 15% throughout the world [1,2]. A recent review estimated that preterm birth affects approximately 11% of births worldwide [3]. In Thailand, the incidence of preterm deliveries has been reported to be 9%-13% with an upward trend [4,5].

Preterm delivery contributed to an increased risk of perinatal and neonatal mortality and morbidity. Incomplete organogenesis and functional development in preterm delivery result in respiratory distress, compromised immune system, visual disabilities, intraventricular hemorrhage, and cardiovascular diseases such as pulmonary hypertension [1-3]. Therefore, prevention of premature delivery is crucial to decrease the risk of mortality and morbidity in neonates. Four categories of preterm delivery have been reported, i.e., spontaneous unexplained preterm labor with intact membranes, premature preterm rupture of membranes, indicated preterm birth due to maternal conditions, and multifetal pregnancy. The first two categories are classified as “spontaneous preterm delivery” and accounted for the major cause of preterm birth [3].

A systematic meta-analysis reported associated risk factors for preterm delivery, including history of preterm birth, maternal age below 19 years, low educational status, sexually transmitted disease, short cervical length of <2.5 cm, smoking, pregnancy by in vitro fertilization (IVF), and pregnancy with complication such as preeclampsia and gestational diabetes mellitus (GDM) [3]. However, the majority of preterm births occur in women without a clear risk factor.

GDM is one of the most common complications in pregnancy [6,7]. It is estimated that 16.2% of alive neonates throughout the world were given birth by women with high blood sugar level, of which 86.4% had GDM [8]. Among other maternal and neonatal complications, preterm delivery has been reported to increase in women with GDM but with conflicting results. Some previous studies demonstrated that pregnancy complicated with GDM or overt diabetes was at a higher risk of preterm delivery compared to pregnancy with normoglycemia [9-11], while others did not find such association [12].

As both GDM and preterm delivery have increased during the past many years in Siriraj Hospital, the primary objective of this study was to evaluate the rate of spontaneous preterm delivery between GDM and normal pregnancy. Secondary objective was to evaluate pregnancy outcomes between the two groups as well as associated risk factors for spontaneous preterm delivery.

## Materials and methods

A retrospective cohort study was conducted at the Department of Obstetrics and Gynecology, Faculty of Medicine, Siriraj Hospital, after approval from Siriraj Institutional Review Board. A total of 600 singleton pregnant women who started antenatal care before 24 weeks of gestation and received GDM screening according to the institutional guideline during June to December 2020 were included. Pregnant women with pre-pregnancy diabetes, chronic underlying diseases such as chronic hypertension, cardiovascular disease, or pulmonary disease were excluded. The sample size was calculated based on the estimated incidence of spontaneous preterm delivery of 20% in GDM and 10% in those without GDM. At 95% confidence level and 80% power with 1:4 ratio, at least 115 women with GDM and 460 without GDM were required, including 10% loss. In this study, 120 women with GDM and 480 women randomly selected from those without GDM during the same period of the study were included as study and comparison groups, respectively.

All women were screened for GDM using a 50-g glucose challenge test (GCT) with 140-mg/dL cutoff value, and a 100-g oral glucose tolerance test was used as a diagnostic test with Carpenter and Coustan criteria. All women received GDM screening during the first antenatal care visit, and repeated tests were offered during 24-28 weeks of gestation if initial tests were normal. After diagnosis of GDM, nutritional counseling was provided together with advice on behavioral modification. A 2-hour postprandial and/or fasting plasma glucose was used for follow-up of glycemic control, either by intermittent testing or self-monitoring blood glucose. Well glycemic control was considered when >70% of fasting plasma or 2-hour postprandial glucose level was within glycemic target (<95 and <120 mg/dL, respectively). Insulin therapy was initiated as appropriate when the women had poor glycemic control after nutritional therapy. According to the institutional guideline, HbA1c was not used either for diagnosis or follow-up of GDM.

In terms of preterm risk factors, history of previous preterm births was routinely asked and recorded during the first antenatal care visit. In addition, all women were offered cervical length measurement during 14-16 weeks. All women with any risk factors for preterm birth (previous preterm birth or cervical length of <2.5 cm) received progesterone treatment in various forms (oral, vaginal, or intramuscular route) to reduce the risk of preterm births.

Data were retrieved from medical records and included baseline characteristics, obstetric history, preterm risks, GDM risks and screening results, data on delivery, and pregnancy and neonatal outcomes. GDM risks included age ≥ 30 years, family history of DM, body mass index (BMI) of ≥25 kg/m2, previous GDM, previous macrosomia, previous unexplained fetal death, and hypertension. Preterm risks included previous preterm birth and short cervix (cervical length of <2.5 cm). Spontaneous preterm birth was defined as delivery before 37 completed weeks of gestation that had been preceded by spontaneous labor. Deliveries by induction of labor or pre-labor cesarean section due to maternal or fetal indications were considered as indicated preterm birth [1].

Data were analyzed with descriptive statistics and presented as mean, standard deviation, number, and percentage as appropriate. Various characteristics and incidence of spontaneous preterm birth were compared between those with and without GDM using Student’s t-test and chi-square test as appropriate. A multivariate logistic regression analysis was used to determine if GDM was independently associated with spontaneous preterm delivery, adjusted for potential confounders. Adjusted odds ratio (OR) and 95% confidence intervals (CI) were estimated. A value of <0.05 was considered statistically significant.

This article was previously posted to the medRxiv preprint server on April 27, 2020.

## Results

Of the 600 singleton pregnancies enrolled in the study, the study group included 120 pregnant women diagnosed with GDM and the comparison group included 480 pregnant women without GDM. Table 1 shows the comparison of various characteristics between the two groups. Both groups were comparable in terms of age, parity, and previous abortion. Compared with those without GDM, women with GDM were more likely to be 30 years of age (65% vs. 54.2%, p=0.032) and have previous GDM (3.3% vs. 0.6%, p=0.013). Both groups were comparable with regard to preterm risks, including previous preterm birth and short cervix. Among GDM cases, six (5%) women required insulin therapy. All GDM cases had well glycemic control.

**Table 1:** Comparison of baseline characteristics between the two groups GDM, gestational diabetes mellitus

Characteristics	No GDM (n=480)	GDM (n=120)	P-value
Mean age ± SD (years)	30.2 ± 5.8	31.8 ± 5.3	0.006
Mean BMI ± SD (kg/m^2^)	22.5 ± 4.6	23.3 ± 3.9	0.094
Nulliparous	221 (46%)	49 (40.8%)	0.305
Previous abortion	97 (20.2%)	24 (20.0%)	0.959
GDM risks			
Age ≥ 30 years	260 (54.2%)	78 (65%)	0.032
Family history of DM	81 (16.9%)	30 (25%)	0.110
BMI ≥ 25 kg/m^2^	112 (23.3%)	33 (27.5%)	0.340
Previous GDM	3 (0.6%)	4 (3.3%)	0.013
Previous macrosomia	7 (1.5%)	3 (2.5%)	0.425
Previous unexplained fetal death	5 (1.0%)	4 (3.3%)	0.065
Hypertension	11 (2.3%)	2 (1.7%)	0.674
Preterm risks			
Previous preterm birth	30 (6.3%)	12 (10.0%)	0.150
Short cervix	2 (0.4%)	1 (0.8%)	0.563
Insulin therapy	-	6 (5.0%)	-

Maternal outcomes were compared between the two groups, and the results are shown in Table 2. While GA at delivery was comparable, incidence of overall preterm delivery was significantly higher in GDM than those without GDM (17.5% vs. 8.5%, p=0.004). Likewise, the incidence of spontaneous preterm delivery was also significantly higher among GDM women (15.8% vs. 7.1%, p=0.004). Rates of indicated preterm were comparable between the two groups (1.5% vs. 1.7%, p=0.866). All cases with indicated preterm births were complicated with preeclampsia. Gestational weight gain (differences between weight at delivery and pre-pregnancy weight) was significantly lower among GDM women (p<0.001) and they were less likely to have excessive weight gain (30% vs. 42.3%, p=0.002). There was no difference in route of delivery and cesarean section indications. Rates of preeclampsia and postpartum hemorrhage were not significantly different between the two groups.

**Table 2:** Comparison of maternal outcomes between the two groups ^1^Normal GWG is 11.5-16 kg for normal BMI, 12.5-18 kg for underweight, 7-11.5 kg for overweight, and 5-9 kg for obese. Inadequate and excessive GWG is those with weight gain below and above normal range, respectively. ^2^Cases included breech presentation, preeclampsia, and placenta previa. GA, gestational age; GDM, gestational diabetes mellitus; GWG, gestational weight gain

Maternal outcomes	No GDM (n=480)	GDM (n=120)	P-value
Mean GWG ± SD (kg)	14.12 ± 5.89	11.99 ± 5.90	<0.001
GWG category^1^			0.002
Normal	156 (32.5%)	35 (29.2%)	
Inadequate weight gain	121 (25.2%)	49 (40.8%)	
Excessive weight gain	203 (42.3%)	36 (30.0%)	
Mean GA at delivery ± SD (weeks)	38.2 ± 1.5	37.9 ± 1.6	0.263
Preterm delivery	41 (8.5%)	21 (17.5%)	0.004
Spontaneous preterm birth	34 (7.1%)	19 (15.8%)	0.004
Indicated preterm birth	7 (1.5%)	2 (1.7%)	0.867
Route of delivery			0.369
Vaginal delivery	270 (56.3%)	61 (50.8%)	
Primary cesarean section	135 (28.1%)	34 (28.3%)	
Repeat cesarean section	75 (15.6%)	25 (20.8%)	
Cesarean section indications			0.902
Cephalopelvic disproportion	53 (39.3%)	14 (41.2%)	
Non-reassuring fetal heart rate	11 (8.1%)	2 (5.9%)	
Others^2^	71 (52.6%)	18 (52.9%)	
Preeclampsia	17 (3.5%)	7 (5.8%)	0.256
Postpartum hemorrhage	29 (6.0%)	13 (10.8%)	0.068

Table 3 shows comparison of neonatal outcomes between the two groups. While mean birth weight was not significantly different, rates of large for gestational age (LGA) and macrosomia were significantly higher among GDM women (24.2% vs. 15.4%, p=0.02, and 5.8% vs. 2.1%, p=0.027, respectively). Neonatal hypoglycemia was significantly more common in those with GDM than those without GDM (7.5% vs. 2.7%, p=0.013). Other neonatal outcomes were comparable, including an Apgar score of <7 at 1 and 5 minutes, phototherapy, and NICU admission. Further analyses were performed, comparing neonatal outcomes between preterm births in GDM and non-GDM women. The results revealed that all neonatal outcomes of these preterm infants were comparable between the two groups, including GA at delivery, birth weight, Apgar scores, hypoglycemia, phototherapy, and NICU admission.

**Table 3:** Comparison of neonatal outcomes between the two groups AGA, appropriate for gestational age; LGA, large for gestational age; SGA, small for gestational age

Neonatal outcomes	No GDM (n=480)	GDM (n=120)	P-value
Mean birth weight ± SD (g)	3057.4 ± 423.3	3110.8 ± 553.8	0.325
Birth weight for GA			0.020
AGA	382 (79.6%)	81 (67.5%)	
SGA	24 (5.0%)	10 (8.3%)	
LGA	74 (15.4%)	29 (24.2%)	
Macrosomia	10 (2.1%)	7 (5.8%)	0.027
Apgar score <7			
1 minute	17 (3.5%)	6 (5.0%)	0.462
5 minutes	1 (0.2%)	0 (0.0%)	0.616
Phototherapy	80 (16.7%)	20 (16.7%)	0.985
Hypoglycemia	13 (2.7%)	9 (7.5%)	0.013
NICU admission	10 (2.1%)	3 (2.5%)	0.784

Multivariate logistic regression analysis was performed to determine if GDM was independently associated with spontaneous preterm birth, adjusted for potential confounders, and the results are shown in Table 4. The significant independent risk factors associated with spontaneous preterm delivery were previous preterm birth (adjusted OR: 2.56, 95% CI: 1.13-5.79, p=0.024) and GDM (adjusted OR: 2.15, 95% CI: 1.2-3.84, p= 0.01).

**Table 4:** Logistic regression analysis to determine the independent risk factor for preterm birth adjusted for potential confounders BMI, body mass index; GDM, gestational diabetes mellitus

Risk factors	Adjusted OR (95% CI)	P-value
Nulliparous	1.01 (0.56-1.78)	0.990
Previous abortion	0.88 (0.44-1.77)	0.722
Age ≥ 30 years	0.93 (0.53-1.63)	0.796
BMI ≥ 25 kg/m^2^	1.51 (0.84-2.72)	0.168
Previous preterm birth	2.56 (1.13-5.79)	0.024
GDM	2.15 (1.20-3.84)	0.010

## Discussion

Preterm delivery commonly causes neonatal morbidities and mortality. Prematurely born neonates are prone to various serious medical complications both early and later in life, including respiratory distress, bronchopulmonary dysplasia, pneumothorax, pneumonia, sepsis, patent ductus arteriosus, necrotizing enterocolitis, intraventricular hemorrhage, and cerebral palsy [1-3]. Therefore, understanding the nature of the condition and associated risks might help in better prevention of preterm delivery and improving pregnancy outcomes.

While mean GA at delivery was comparable between the two groups, incidence of spontaneous preterm delivery in this study was significantly higher in GDM compared to non-GDM group (15.8% vs. 7.1%, p=0.004), and multivariate analysis showed that GDM independently increased the risk of preterm birth by 2.15 times (95% CI: 1.20-3.84). The findings were similar to other previous studies [9-11]. Another study showed a similar risk of preterm birth between those with and without GDM [13]. Variations in the results between studies might partly be due to differences in population characteristics, GDM screening and risks, risk of preterm delivery, and associated management scheme. Some previous studies also reported increased risk of preterm delivery as well as other adverse outcomes among GDM women that were detected early in pregnancy despite treatment [14,15]. This could possibly be related to the higher severity of hyperglycemia among this specific group of women. Higher risk of preterm birth was also observed in women with insulin-treated GDM than those with diet-treated GDM in a previous study [11]. However, the number of insulin-treated cases in this study was too small to evaluate such a relationship with valid and reliable results, and further studies might be needed. The exact mechanism for the observed association was still not known. Further biologic explanations of a possible relationship between GDM and spontaneous preterm delivery are to be explored.

Similar to current knowledge and many previous studies [1-3,16,17], the results of this study showed that previous preterm birth was the other strong independent associated factor for preterm birth (adjusted OR: 2.56). Progesterone has been demonstrated to be effective in decreasing the risk of preterm birth among this at-risk population [18-20]. As of the institutional guideline, all women at a high risk for preterm delivery were offered progesterone treatment for the prevention of preterm birth, either via vaginal, oral, or intramuscular route. However, GDM is not currently included as one of the risks for preterm delivery. If, in the future, evidence on association between GDM and preterm birth is documented, administration of progesterone might have some role in preterm prevention among this group of women.

Other pregnancy outcomes related to GDM were as expected and have been repeatedly reported [6]. Lower gestational weight gain among GDM has been consistently reported from the same institution [21,22]. This was probably due to the effect of intensive individual counseling on each GDM woman as well as close monitoring. Increased LGA as well as macrosomia among GDM women were also similar to previous studies [21-23]. Clinical risks associated with GDM were similar to what has been reported from many previous studies, including increasing age and BMI, and previous GDM [24,25].

The results of this study also emphasize the importance of early diagnosis and good management of GDM, which could also reduce the risk of preterm birth. Additional use of some markers from the first trimester can also facilitate GDM diagnosis and could also reduce the risk of preterm birth and its related adverse outcomes [26,27].

The strength of this study may include the uniform diagnosis of GDM, under the same institutional guideline. Samples were randomly selected from the same population. Selection bias should be minimal as shown by the rate of preterm delivery among non-GDM women, which was relatively similar to those of other low-risk women in the same hospital. Some limitations should be mentioned. Data were from a tertiary care hospital, which could have limited the generalizability of the results. In addition, there were variations on GDM screening strategy as well as preterm management between settings due to which the results might not be applicable. There were too small numbers of cases in some subgroups of GDM (insulin-treated group or early GDM group) to further evaluate the differences in rates of preterm births with valid and reliable results.

Whether GDM and preterm birth are strongly related needs to be confirmed by future studies. Possible biologic explanations should also be explored. If more strong evidence can be demonstrated in the future, a prevention program among this group of women should be developed in order to minimize neonatal morbidities and mortality associated with preterm birth.

## Conclusions

Women with GDM are at a significantly increased risk of spontaneous preterm delivery. The other significant risk factor was previous preterm birth. GDM also increased the risk of some adverse outcomes, including LGA, macrosomia, and neonatal hypoglycemia.
